# Inverse Association Between Serum Vitamin B12 Concentration and Obesity Among Adults in the United States

**DOI:** 10.3389/fendo.2019.00414

**Published:** 2019-06-27

**Authors:** Yangbo Sun, Minxian Sun, Buyun Liu, Yang Du, Shuang Rong, Guifeng Xu, Linda G. Snetselaar, Wei Bao

**Affiliations:** ^1^Department of Epidemiology, College of Public Health, University of Iowa, Iowa City, IA, United States; ^2^Department of Endocrinology, Wuhan Central Hospital Affiliated to Tongji Medical College, Huazhong University of Science and Technology, Wuhan, China; ^3^Obesity Research and Education Initiative, University of Iowa, Iowa City, IA, United States; ^4^Fraternal Order of Eagles Diabetes Research Center, University of Iowa, Iowa City, IA, United States

**Keywords:** vitamin B12, nutrition, micronutrient, obesity, adults

## Abstract

**Introduction:** Several studies have suggested that vitamin B12 deficiency is more common in obese individuals. We evaluated the cross-sectional associations of serum vitamin B12 concentrations with obesity in a nationally representative sample of adults in the United States.

**Methods:** We included 9,075 participants aged ≥20 years in the National Health and Nutrition Examination Survey 2011–2014. Serum vitamin B12 concentrations were measured by electrochemiluminescence immunoassay. Obesity was defined as BMI ≥30 kg/m^2^. We used logistic regression with sample weights to estimate the odds ratios (ORs) and 95% confidence intervals (CIs).

**Results:** Serum vitamin B12 concentrations were lower among obese adults compared with non-obese adults. After adjustment for age, gender, race/ethnicity, socioeconomic status, dietary and lifestyle factors, use of medications that could affect the serum vitamin B12 levels, dietary supplement use and fasting time, the multivariable-adjusted ORs (95% CIs) of obesity were 1.00 (reference), 0.95 (0.79, 1.14), 0.86 (0.74, 0.99), and 0.71 (0.60, 0.84) (*p* for trend <0.001) for increasing quartiles of serum vitamin B12 concentrations.

**Conclusions:** In a large nationally representative sample of U.S. adults, higher serum vitamin B12 levels were inversely associated with obesity. Further investigation is needed to understand the underlying mechanisms.

## Introduction

Obesity has become one of the most costly chronic disorders worldwide, and it largely explains the dramatic increase in the incidence and prevalence of type 2 diabetes and cardiovascular diseases over the past 20 years (1, 2). It is estimated that obesity affects 500 million adults worldwide (3). Data from the National Health and Nutrition Examination Survey 2015–2016 show that roughly 2 out of 5 U.S. adults are obese (4). Clearly, obesity is a disorder of the energy homeostasis system, and dietary compositions can theoretically affect energy balance by altering overall energy intake, energy expenditure, or both (5).

Vitamin B12 is essential for one-carbon metabolism. It is involved in cellular energy production and epigenetic modulation processes, including DNA methylation, synthesis, and repair (6). Vitamin B12 is concentrated in animal tissues, hence, vitamin B12 is found only in foods of animal origin. Foods that are high in vitamin B12 include liver, beef, lamb, chicken, eggs and dairy foods (7). Vitamin B12 deficiency is mainly due to limited dietary intake of animal foods or malabsorption of the vitamin (8). Several studies have suggested that vitamin B12 deficiency is more common in obese individuals, such as obese children and adolescents (9), obese women with polycystic ovary syndrome (10), and obese pregnant women (11–13). However, findings from previous studies on the association between serum vitamin B12 concentrations and obesity have been inconsistent (11–18). In this study, we evaluated the associations of serum vitamin B12 concentrations with obesity in a large, nationally representative sample of U.S. adults.

## Materials and Methods

### Study Population

The study population consisted of participants from the 2011–2012 and 2013–2014 cycles of the National Health and Nutrition Examination Survey (NHANES). Briefly, the NHANES is a large-scale, ongoing, nationally representative health survey of the non-institutionalized US population. It is conducted by the National Center for Health Statistics (NCHS) of the Centers for Disease Control and Prevention (CDC). NHANES survey data are released every 2 years; each cycle consists of ~10,000 participants (19). The data are comprised of population-based, cross-sectional surveys about diet, nutritional status, general health, disease history, and health behaviors (19). The surveys use multi-stage, probability clusters to develop a population sample that is nationally representative of the U.S. based on age, sex, and race/ethnicity. NHANES data along with documents on the survey methods and other information are publicly available on the NHANES online website. The study protocol was approved by the NCHS Research Ethics Review Board (#2011–17). All subjects gave written informed consent.

For this analysis, we initially identified 10,066 participants aged 20 years or older, with complete information on serum vitamin B12 levels and obesity status. We then excluded 876 women who had history of cancer or malignancy, 108 women who were currently pregnant and 7 women who had missing information on smoking status, resulting in 9,075 participants being finally included in our analysis.

### Exposure Measurement

Serum vitamin B12 was measured in adults 20 years and older using the fully automated electrochemiluminescence immunoassay on the Roche Elecsys 170 System (Roche Diagnostics, Indianapolis, IN). Vials were stored under appropriate frozen (−20°C) conditions until they were shipped to National Center for Environmental Health for testing. The lower limit of detection (LLOD) for vitamin B12 was 30 pg/mL (i.e., 22.14 pmol/L). The coefficient of variation for this assay was lower than 4%.

### Outcome Measurement

For all surveys, weight and height were measured in a mobile examination center using standardized techniques and equipment by trained health technicians according to the NHANES Anthropometry Procedures Manual (20). Body mass index (BMI) was calculated as weight in kilograms divided by the square of height in meters, and rounded to 1 decimal place. Obesity was defined as BMI ≥30 kg/m^2^.

### Covariate Assessment

Information on age, gender, race/ethnicity, education, annual household income, family history of diabetes, smoking status, physical activity, and use of medications was collected during the interview (21). Race/ethnicity was categorized as non-Hispanic white, non-Hispanic black, Hispanic (Mexican and non-Mexican Hispanic), and other race/ethnicity. Education was grouped as less than high school, high school, and college or higher. Family income-to-poverty ratios were categorized as ≤1.30, 1.31–3.50, and >3.50 (22). Individuals who smoked <100 cigarettes in their lifetime were defined as never smokers; those who had smoked more than 100 cigarettes but did not smoke at the time of survey were considered former smokers; those who had smoked more than 100 cigarettes and smoked cigarettes at the time of survey were current smokers (23). NHANES examinations took place in the mobile examination center (MEC). During the MEC exam, a 24-h dietary recall is administered by trained dietary interviewers. Each MEC dietary interview room contains a standard set of measuring guides to help the respondent report the volume and dimensions of the food items consumed and estimate portion sizes. Beginning in 2002, all participants were asked to complete a second 24-h dietary recall (Day 2) interview, collected by telephone ~3–10 days after the MEC exam. Upon completion of the in-person interview, participants are given measuring cups, spoons, a ruler, and a food model booklet, which contain two-dimensional drawings of the various measuring guides available in the MEC, to use for reporting food amounts during the telephone interview. Dietary intake was assessed through two 24-h dietary recalls. Total energy intake, and alcohol intake, were calculated using a food composition database (24, 25). Alcohol intake was categorized as non-drink (0 g/day), moderate drinking (0.1–27.9 g/day for men and 0.1–13.9 g/day for women), and heavy drinking (≥28 g/day for men and ≥14 g/day for women) (26). Physical activity was assessed using the Global Physical Activity Questionnaire. We classified physical activity into three groups (<600, ≥600–1,199, and ≥1,200 metabolic equivalent [MET]-min/week) (27). Use of medication that could have an effect on serum vitamin B12 was defined as use of any of the following: metformin, histamine-2 blockers (H2-blockers) or proton inhibitors (PPIs) (28–30). Dietary supplement use was defined as use of any vitamins, minerals or other dietary supplements in the past month. Fasting time (in hours) was calculated from when the examinee last ate or drank anything other than water to the time of the venipuncture.

### Statistical Analysis

All statistical analyses accounted for the complex, multistage, stratified, cluster-sampling design of NHANES by using sample weights, strata, and primary sampling units embedded in the NHANES data. Comparisons of characteristics across serum vitamin B12 levels were performed using analysis of variance for continuous variables and chi-square test for categorical variables.

We used multivariable logistic regression to estimate odds ratios (ORs) and 95% confidence intervals (CIs) of obesity risk according to quartiles of serum vitamin B12 concentrations. In multivariable models, we adjusted for age, gender, race/ethnicity, education, family income to poverty ratio, smoking status, alcohol intake, physical activity, dietary vitamin B12 intake, total energy intake, use of metformin, H2-blockers and PPIs, dietary supplement use, and fasting time.

We evaluated if the associations varied with gender (male and female), and race/ethnicity (white, and non-white [non-Hispanic black, Hispanic and others]). We conducted interaction tests via multiplicative interaction terms in the multivariable models. We also conducted sensitivity analyses (1) excluding participants with abnormal serum vitamin B12 levels (<200 pg/ml) (31); (2) excluding participants with fasting time <8 h. All analyses were performed using survey procedures in SAS 9.4 (SAS Institute, Cary, NC).

## Results

We included 9,075 participants in this study with an average age of 45.9 years [standard error (SE) = 0.45]. The weighted prevalence of overweight was 33.5% (SE = 0.76), and the weighted prevalence of obesity was 36.6% (SE = 0.86). Individuals with higher serum vitamin B12 levels were more likely to be non-white, non-smoking, to have less alcohol intake, to have higher dietary vitamin B12 intake, to have dietary supplements, and to have a shorter fasting time (Table 1).

**Table 1:** Characteristics of the study population (*n* = 9,075), according to quartiles of serum vitamin B12 concentrations.

	**Serum vitamin B12 concentrations, pg/ml**				
Characteristic	Quartile 1 (<386.0)	Quartile 2 (386.0–521.0)	Quartile 3 (522.0–719.0)	Quartile 4 (≥720.0)	*P*-value
No. of participants	2,262	2,281	2,264	2,268	
Age, years	45.9 (0.52)	44.5 (0.59)	44.4 (0.63)	48.9 (0.47)	<0.001
**Gender, %**					
Male	49.1 (1.49)	51.5 (0.94)	52.2 (1.38)	44.1 (1.12)	<0.001
Female	50.9 (1.49)	48.5 (0.94)	47.8 (1.38)	55.9 (1.12)	
**Race/ethnicity, %**					
Non-Hispanic white	68.2 (2.47)	66.1 (2.93)	63.1 (2.85)	61.5 (2.88)	<0.001
Non-Hispanic black	14.2 (1.86)	16.2 (2.09)	17.1 (1.91)	14.6 (1.83)	
Hispanic	9.6 (1.29)	10.6 (1.32)	11.8 (1.49)	13.9 (1.71)	
Other	8.1 (0.96)	7.0 (0.61)	8.0 (0.84)	10.1 (1.12)	
**Education, %**					
Less than high school	16.0 (1.27)	16.6 (1.44)	16.1 (1.38)	16.2 (1.53)	0.99
High school	21.2 (1.65)	21.3 (1.37)	21.0 (1.31)	20.9 (1.04)	
College or above	62.8 (2.00)	62.1 (2.14)	63.0 (1.97)	62.9 (1.93)	
**Ratio of family income to poverty, %**					
≤1.30	25.4 (1.64)	25.2 (1.54)	23.6 (1.79)	21.5 (1.71)	0.17
1.31–3.50	32.4 (1.52)	31.5 (1.46)	30.6 (1.60)	32.8 (1.65)	
>3.50	36.5 (2.22)	37.9 (2.47)	38.9 (2.16)	39.1 (2.41)	
Missing	5.8 (0.71)	5.4 (0.57)	6.8 (0.79)	6.6 (0.78)	
**Smoking status, %**					
Non-smoker	55.7 (1.30)	56.1 (1.03)	56.4 (1.41)	61.0 (1.64)	<0.001
Current smoking	22.6 (1.48)	22.5 (1.13)	19.5 (1.26)	15.0 (0.89)	
Ever smoker	21.7 (1.13)	21.4 (1.32)	24.1 (1.00)	24.2 (1.36)	
**Alcohol intake^a^, %**					
Non-drinker	67.6 (1.63)	64.1 (1.96)	66.8 (1.73)	70.8 (1.33)	0.01
Moderate drinking	7.2 (0.74)	9.3 (0.75)	6.7 (0.75)	7.2 (0.89)	
Heavy drinking	19.3 (1.21)	20.6 (1.51)	20.2 (1.54)	15.0 (1.19)	
Missing	5.8 (0.70)	5.9 (0.74)	6.5 (0.79)	7.0 (0.64)	
Dietary vitamin B12 intake, mcg	4.1 (0.11)	5.4 (0.39)	5.6 (0.13)	5.8 (0.15)	<0.001
**Physical activity, MET-min/wk**					
<600	37.2 (1.33)	34.6 (1.54)	31.7 (0.95)	38.2 (1.47)	0.01
≥600–1,199	11.3 (0.77)	10.3 (0.92)	11.0 (0.76)	11.4 (1.04)	
≥1,200	51.5 (1.39)	55.1 (1.76)	57.3 (1.06)	50.4 (1.71)	
Total energy intake (kcal/day)	2,190 (29.2)	2,234 (20.9)	2,275 (17.3)	2,146 (23.9)	<0.001
Use of metformin, H2-blockers or PPIs, %	14.5 (1.02)	14.4 (1.15)	11.6 (1.01)	19.0 (1.10)	<0.001
**Dietary supplement use, %**					
Yes	37.6 (1.2)	44.1 (1.8)	53.5 (1.7)	69.4 (1.5)	<0.001
No	62.4 (1.2)	55.9 (1.8)	46.5 (1.7)	30.6 (1.5)	
Fasting hours	7.4 (0.2)	7.0 (0.2)	6.9 (0.1)	6.8 (0.2)	0.02
BMI, kg/m^2^	29.6(0.22)	29.3(0.22)	28.7(0.18)	28.1(0.23)	<0.001

*Values are means (SE) for continuous variables or percentages (SE) for categorical variables and are weighted expect No. of participants. MET, metabolic equivalent; H2, blockers, histamine-2 blockers; PPIs, proton pump inhibitors*.

a *Non-drinker: 0 g/day; Moderate drinking: 0.1–28 g/day for men and 0.1–14 g/day for women; Heavy drinking: ≥ 28 g/day for men and ≥ 14 g/day for women*.

Serum vitamin B12 concentrations were lower among obese adults compared with non-obese adults. After adjustment for age, gender, race/ethnicity, socioeconomic status, dietary and lifestyle factors, use of medications that could affect the serum vitamin B12 levels, dietary supplements use and fasting time, the multivariable-adjusted ORs (95% CIs) of obesity were 1.00 (reference), 0.95 (0.79, 1.14), 0.86 (0.74, 0.99), and 0.71 (0.60, 0.84) (*p* for trend <0.001) for increasing quartiles of serum vitamin B12 concentrations (Table 2).

**Table 2:** Associations of serum vitamin B12 concentrations with obesity in 9,075 US adults.

	**Serum vitamin B12 concentrations, pg/ml**				
	Quartile 1 (<386.0)	Quartile 2 (386.0–521.0)	Quartile 3 (522.0–719.0)	Quartile 4 (≥720.0)	*P* for trend
No. of participants	2,262	2,281	2,264	2,268	
Model 1^b^	1 (ref)	0.94 (0.79, 1.11)^a^	0.82 (0.73, 0.94)	0.71 (0.61, 0.84)	<0.001
Model 2^c^	1 (ref)	0.93 (0.78, 1.11)	0.81 (0.70, 0.94)	0.67 (0.57, 0.79)	<0.001
Model 3^d^	1 (ref)	0.95 (0.79, 1.14)	0.86 (0.74, 0.99)	0.71 (0.60, 0.84)	<0.001

a *OR; 95% CI in parentheses (all such values)*.

b *Model 1: adjusted for age and gender*.

c *Model 2: Model 1 plus race/ethnicity, education, family income, cigarette smoking, physical activity, alcohol intake, dietary vitamin B12 intake, and total energy intake, as categorized in Table 1*.

d *Model 3: Model 2 plus use of metformin, histamine-2 blockers or proton pump inhibitors, dietary supplement use and fasting time*.

These associations remained the same in sensitivity analysis restricting to participants with normal serum vitamin B12 levels (Table 3). Individuals with the highest serum vitamin B12 levels were less likely to be obese, with the multivariable-adjusted OR (95% CI) as 0.71 (0.60, 0.84), compared with individuals with the lowest serum vitamin B12 level. These associations appeared more profound in sensitivity analysis restricting to participants with fasting time more than 8 h (Table 4). Individuals with the highest serum vitamin B12 levels were less likely to be obese, with the multivariable-adjusted OR (95% CI) of obesity as 0.65 (0.51, 0.83), compared with individuals with the lowest serum vitamin B12 level. The association of serum vitamin B12 levels with obesity did not vary according to gender or race/ethnicity (*p* ≥ 0.12).

**Table 3:** Sensitivity analysis for the association of serum vitamin B12 concentrations with obesity in 8,903 US adults with normal vitamin B12 concentrations.

	**Serum vitamin B12 concentrations, pgl/ml**				
	Quartile 1 (200.0–393.0)	Quartile 2 (394.0–526.0)	Quartile 3 (527.0–723.0)	Quartile 4 (≥724.0)	*P* for trend
No. of participants	2,224	2,230	2,222	2,227	
Model 1^b^	1 (ref)	0.90 (0.77, 1.06)^a^	0.82 (0.72, 0.93)	0.71 (0.60, 0.84)	0.001
Model 2^c^	1 (ref)	0.91 (0.77, 1.08)	0.80 (0.69, 0.94)	0.67 (0.57, 0.79)	<0.001
Model 3^d^	1 (ref)	0.92 (0.77, 1.10)	0.84 (0.73, 0.98)	0.71 (0.60, 0.84)	<0.001

a *OR; 95% CI in parentheses (all such values)*.

b *Model 1: adjusted for age and gender*.

c *Model 2: Model 1 plus race/ethnicity, education, family income, cigarette smoking, physical activity, alcohol intake, dietary vitamin B12 intake, and total energy intake, as categorized in Table 1*.

d *Model 3: Model 2 plus use of metformin, histamine-2 blockers or proton pump inhibitors, dietary supplement use and fasting time*.

**Table 4:** Sensitivity analysis for the association of serum vitamin B12 concentrations with obesity in 4,567 US adults fasting more than 8 h.

	**Serum vitamin B12 concentrations, pgl/ml**				
	Quartile 1 (<380.0)	Quartile 2 (380.0–512.0)	Quartile 3 (513.0–700.0)	Quartile 4 (≥701.0)	*P* for trend
No. of participants	1,135	1,150	1,141	1,141	
Model 1^b^	1 (ref)	0.91 (0.74, 1.13)^a^	0.80 (0.67, 0.96)	0.68 (0.54, 0.86)	<0.001
Model 2^c^	1 (ref)	0.87 (0.71, 1.08)	0.79 (0.66, 0.95)	0.64 (0.51, 0.80)	<0.001
Model 3^d^	1 (ref)	0.89 (0.72, 1.11)	0.81 (0.67, 0.98)	0.65 (0.51, 0.83)	<0.001

a *OR; 95% CI in parentheses (all such values)*.

b *Model 1: adjusted for age and gender*.

c *Model 2: Model 1 plus race/ethnicity, education, family income, cigarette smoking, physical activity, alcohol intake, dietary vitamin B12 intake, and total energy intake, as categorized in Table 1*.

d *Model 3: Model 2 plus use of metformin, histamine-2 blockers or proton pump inhibitors, dietary supplement use and fasting time*.

## Discussion

Based on nationally representative data, we found an inverse and significant association between serum vitamin B12 levels and obesity in a dose-response manner. Compared with individuals with higher vitamin B12 concentrations, those with lower vitamin B12 concentrations were more likely to be obese. These associations were independent of demographic, socioeconomic, lifestyle factors, use of medications that could have an effect on serum vitamin B12 levels, dietary supplement use and fasting time.

To our knowledge, this is the largest study to date regarding the association of serum vitamin B12 levels with obesity in the general population. Previous studies on the association of serum vitamin B12 levels with obesity in adults have yielded inconsistent findings (11–18). Our results are consistent with some previous studies where serum vitamin B12 levels were inversely associated with obesity (9, 11–13, 15, 16, 32, 33). However, some of the results from those previous studies could not be generalized to other populations because they were restricted to a specific population such as pregnant women (11–13, 32). In addition, our results were consistent with several studies among children and adolescents (16, 33).

There are several possible explanations for these associations. One possible explanation is that low serum vitamin B12 levels would trap folate as 5-methyltetrahydrofolate, prevent the generation of methionine from homocysteine, and therefore reduce protein synthesis and lean tissue deposition (10). It could also be due to the adipocyte dysfunction linked to low vitamin B12 levels through cellular inflammation (13, 34). Another possibility is that obesity could lower serum vitamin B12 levels through decreased dietary intake or absorption, increased catabolism, and sequestration in adipose tissue (12), or changes in the gut microbiota profiles which could affect the metabolism of vitamin B12 (35, 36). Interestingly, a recent study in Danish population showed that lower serum vitamin B12 concentrations were significantly associated higher BMI, but a genetic risk score related to vitamin B12 concentrations associated variants was not associated with BMI (15). The causal relation between vitamin B12 status and obesity warrants further investigation.

The major strength of this population-based study is the use of a nationally representative sample, which facilitates generalization of the findings to the general population in the United States. Other strengths include a large sample size, and a broad range of serum vitamin B12 levels. In addition, with the detailed data collected in the NHANES, we were able to control potential confounding effects from a variety of demographic, socioeconomic, lifestyle factors, use of medications that could have an effect on serum vitamin B12 levels, dietary supplement use and fasting time. Furthermore, the measurement of serum vitamin B12 is not subject to recall bias and thus could provide more reliable results for association studies than dietary estimates. This study has some limitations. First, this study was a cross-sectional survey, thus it could not presume causality. It is difficult to determine if obesity status alters vitamin B12 levels or preexisting low levels of serum vitamin B12 causes obesity, though the inverse association of serum vitamin B12 levels with obesity support the role of B vitamins in adipogenesis. Second, although we have adjusted for demographic, socioeconomic, lifestyle factors and some medications, residual confounding by unmeasured factors is still possible. Third, although we have excluded cancer from the analysis, information of other diseases that may affect vitamin B12 absorbing and body weight, such as atrophic gastritis or pernicious anemia, was not available in NHANES.

## Conclusions

In a nationwide population-based study in the U.S., we showed that individuals with higher serum vitamin B12 levels were less likely to be obese. Further investigation is needed to determine the causality and the underlying mechanisms.

## Ethics Statement

All subjects gave written informed consent. The study protocol was approved by the NCHS Research Ethics Review Board.

## Author Contributions

YS and MS contributed to the design and analysis of the study and wrote the manuscript. WB conceived the idea, interpreted the results, and reviewed and edited the manuscript. BL, YD, SR, GX, and LS interpreted the results and reviewed and edited the manuscript. YS, MS, and WB are the guarantors of this work and, as such, had full access to all the data in the study and take responsibility for the integrity of the data and the accuracy of the data analysis.

### Conflict of Interest Statement

The authors declare that the research was conducted in the absence of any commercial or financial relationships that could be construed as a potential conflict of interest.
